# Impending cauda equina syndrome due to Kummell disease; A case report and literature review

**DOI:** 10.1016/j.ijscr.2021.106041

**Published:** 2021-05-26

**Authors:** Farzad Omidi-Kashani, Ali Parsa, Daniel Madarshahian

**Affiliations:** a Orthopedic Department, Faculty of Medicine, Mashhad University of Medical Sciences, Mashhad, Iran; b The School of Medicine, University of Central Lancashire, Preston, Lancashire, United Kingdom

**Keywords:** Kummell disease, Vertebral fracture, Vertebral osteonecrosis, Case report

## Abstract

**Introduction:**

Kummell disease (KD) is a rare cause of vertebral fracture due to osteonecrosis. The natural history of the disease is characterized by a previous minor trauma, a subclinical window period, and then a symptomatic period presenting with disabling pain, kyphosis, or neurologic deficit.

**Importance:**

As an important but rare cause of non-discogenic cauda equina syndrome.

**Case presentation:**

Here, we report on a wheelchair-bound 28-years-old bodybuilder man with KD who presented with progressive paresthesia and weakness of both legs (impending cauda equina syndrome) due to L5 involvement. He had a past medical history of arbitrary use of licensed and unlicensed drugs in the fitness field. The patient underwent posterior decompression, spinopelvic stabilization, and fusion. Postoperative rehabilitation was satisfactory and after six months, the leg muscle strength returned to normal and the patient's back pain disappeared.

**Conclusion:**

KD should be considered as a rare differential diagnosis when dealing with any patient with a vertebral fracture associated with a history of minor trauma and an asymptomatic window period.

## Highlights

• A case of Kummell disease in a rare location (L5 vertebra) was reported.
• The usual consequence of this disease is a compression fracture in a neurologically intact patient.
• In our case, the creation of a burst fracture induced cauda equina syndrome.
• This case represents a rare cause of non-discogenic cauda equina syndrome.
• Our patient unlike most individuals with Kummell disease, due to significant neurologic deficit needed open surgery.

## 1 Introduction

Kummell disease (KD) is a rare cause of vertebral fracture due to osteonecrosis, but there is no consensus that the etiology of KD is the same as that of femoral head avascular osteonecrosis [1]. The natural history of the disease can be best characterized by a previous minor trauma, a subclinical window period, and then a symptomatic period at which it manifests itself as a progressive disabling pain, kyphosis, or neurologic deficit [2,3]. The disease has some characteristic (non-diagnostic) features on imaging that help to differentiate it from post-traumatic kyphosis, infection, osteoporotic fracture, or metastatic involvement [4,5]. Most symptomatic patients show a satisfactory response to conservative or minimally invasive measures such as percutaneous vertebral body cement augmentation, but in those patients with neurological deficit, open surgery may be indicated [[6], [7], [8], [9], [10], [11]]. Here, we reported a case of a 28 years-old man with KD who presented to us with impending cauda equina syndrome due to L5 involvement which is extremely rare in this disease [12]. This case report has been reported in line with the SCARE Criteria [16].

## 2 Case presentation

A 28-years-old wheelchair-bound man presented to the clinic complaining of severe back pain and an inability to walk for the past two weeks. The patient had no history of actual trauma and had only suffered from minor back pain, after pulling a rug a month prior. Although the pain was initially low in severity, it had gradually increased in intensity and lead to severe disability and progressive weakness in both legs. He also complained of diffuse paresthesia in both legs.

He was a bodybuilder and the only positive finding in his past medical history was the arbitrary use of licensed and unlicensed drugs in the field of bodybuilding. On physical examination, the patient could hardly stand. Manual muscle testing revealed a bilateral weakness in the extensor muscles of the big toe (severe on the left and moderate on the right) and ankle dorsiflexors (moderate on both sides), but sphincter function remained intact. The power of the ankle plantar flexors and quadriceps were also completely normal.

**Fig. 1:**
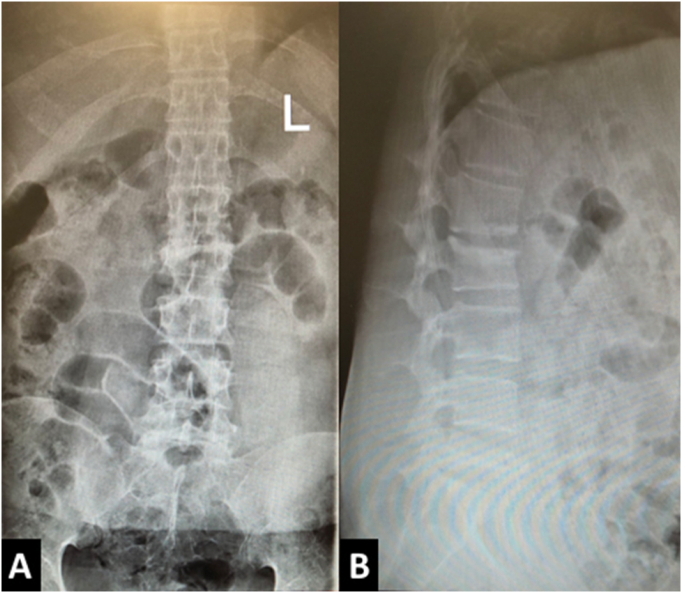
Plain anteroposterior and lateral radiographs of the lumbosacral area showing L2 compression, L5 burst fracture and intervertebral vacuum cleft sign.

**Fig. 2:**
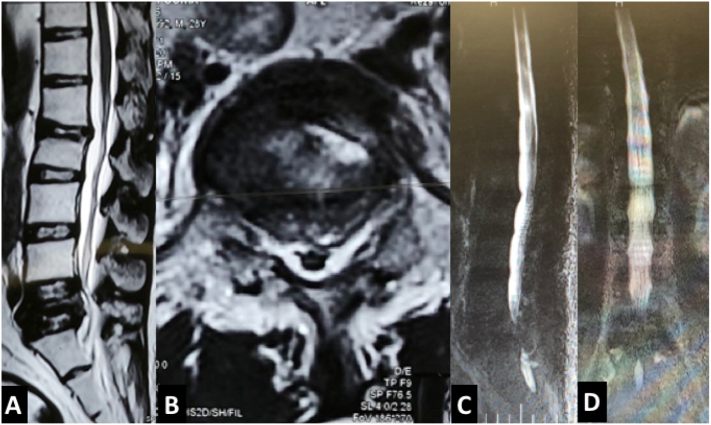
MRI scanning revealed double line sign in the place of 5th vertebral body.

In laboratory testing, a raised C-reactive protein was the only significant finding. In the review of imaging studies, we found a compression fracture in L2 and a burst fracture in L5 with canal compromise and intravertebral vacuum cleft sign in L5 (Fig. 1). Magnetic resonance imaging (MRI) especially on the axial plane showed a double line sign; a linear area of decreased signal intensity (vacuum cleft) within an area of increased signal intensity representing intravertebral fluid (Fig. 2). We could not find any paravertebral soft tissue mass or abscess in this spinal region, and a whole-body bone scan showed isolated increased uptake in only these two areas without any signs of metastatic involvement.

**Fig. 3:**
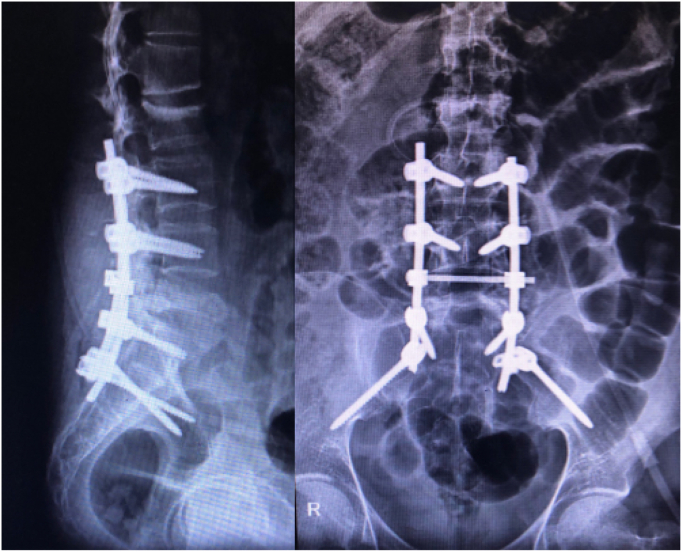
Postoperative anteroposterior and lateral views showed L5 laminectomy, lumbopelvic instrumentation and fusion in appropriate alignment.

Due to the presence of the neurologic deficit (impending cauda equina), we opted not to take a fine needle biopsy, and instead proceeded directly to the surgical intervention (by F.O-K′ surgical team). We placed the patient in the prone position, and a total L5 laminectomy, bilateral foraminotomy, and open bilateral transpedicular biopsy were all carried out along with spinopelvic stabilization and fusion (Fig. 3). On the day after surgery, the patient was mobilized with a soft lumbosacral corset and began lower extremities rehabilitation. Microscopic tissue examination showed fragments of trabecular bony structure with bony sequestrum alongside new bone formation and necrosis, and therefore, KD was confirmed. Six months later, at the latest follow-up visit, his lower back pain and weakness had completely resolved, but he still complained of vague trivial pain and some diffuse paresthesia in both legs which were not interfering with the activities of daily living.

## 3 Discussion

KD is still a disease with many mysterious unknowns. The disease was named after the German surgeon Hermann Kummell in 1895, it was described as a delayed symptomatic vertebral body collapse after a trivial spinal trauma with a subclinical window of about a few weeks or months [13]. KD is not the same as post-traumatic kyphosis, because in the former, the severity of the initial trauma is negligible, and the cause of vertebral body collapse is osteonecrosis, and not simple bone subsidence [14].

There are no clear diagnostic criteria for Kummell disease, and in most cases, it does remain a diagnosis of exclusion. Among the diagnostic criteria stated in the literature for this disease, the following can be mentioned:

1) The characteristic clinical course of the disease: a trivial initial trauma with resultant transient lower back pain usually followed by an asymptomatic window period. Then, a recurring back pain which is usually refractory to conservative measures, and finally a local kyphosis or neurologic involvement may occur [10].
2) Vertebral body contour in serial radiographs: the sequence should show the normal contour of the vertebrae body at first and then vertebral body collapse as the patient becomes symptomatic [10].
3) Intravertebral vacuum cleft sign: although this radiographic sign is indicative of osteonecrosis, it is not pathognomonic and may be seen in an osteoporotic compression fracture, long-term corticosteroid therapy, myeloma, bone metastasis, acute fracture, osteomyelitis, alcoholism, diabetes mellitus, and arteriosclerosis [4,5].
4) Double line sign: as osteonecrosis progresses, the volume of bone decreases and is simultaneously replaced by gas and sometimes fluid which accumulates in this low-pressure area creating a linear hyperintensity surrounded by a hypointense zone of osteonecrosis called double line sign on T2 weighted images. The double line sign on MRI scanning is equal to the intravertebral vacuum cleft sign-on radiography or CT scanning [5].
5) Bone pathology: the pathologic tissue obtained during surgery should reveal ischemic necrosis of the bone [10].

The typical site of involvement is in the thoracic and lumbar regions with T12 being the most commonly affected [12]. Historically, Dr. Steel in 1951 based on clinical characteristics has divided KD into five stages: Stage I, primary trauma with normal radiography; Stage II, initial symptomatic period presenting with trivial back pain; Stage III, asymptomatic interval lasting a few months to years; Stage IV, recurrence stage with progressive pain at the fracture level; and Stage V, terminal stage with resultant kyphosis or neurologic involvement [3]. In later years based on the MRI appearance, the disease was divided into three stages: Stage I, vertebral body height loss <20% with the intact adjacent disc; Stage II, height loss >20% with adjacent degenerative disc disease that reveals dynamic instability on imaging study; and stage III, posterior vertebral body involvement with neurologic compression [2]. Appropriate treatment of KD depends on the patient's staging and clinical complaints. In some cases, conservative treatment including bed rest, flexion control thoracolumbosacral orthosis (TLSO), and analgesics may be sufficient. However, this conservative treatment usually has little effect on relieving pain attributed to the intravertebral instability [15. More aggressive treatment options comprise of nerve root blockage, vertebral body augmentation, and open surgery [6]. In neurologically intact patients with painful vertebral fracture, most authors recommend percutaneous vertebral body augmentation with vertebroplasty or kyphoplasty [[7], [8], [9]]. In these minimally invasive procedures, the unstable vertebra is reconstructed and filed with orthopedic cement and the patient's pain and disability are eliminated immediately after the operation. Even Xia et al. believe that there is a direct correlation between the amount of cement injected and the patient's recovery rate [8].

However, in cases like ours that have significant neurological symptoms, classic surgical treatment is required, even if the underlying osteoporosis is severe [10,11]. In cases with severe underlying osteoporosis, surgical maneuvers (such as longer construct, simultaneous cementing, expandable screws, etc.) should be applied to increase pull-out strength and success rate. Our case report had two new items relative to the existing literature: 1) its rare location in the lower lumbar area (not in a more common location in middle thoracic or upper lumbar), and 2) its rare complication (cauda equina syndrome); usually, KD creates compression fracture that favorably responds to more conservative measures. This is a special case with the creation of a burst fracture in the lower lumbar area, making it quite a rare and fascinating case.

## 4 Conclusion

In conclusion, although KD is a rare cause of back pain, physicians should consider this as part of their differential diagnosis when dealing with any patient with a vertebral fracture associated with a history of minor trauma and an asymptomatic window period.

## Ethical approval

The ethical committee approval was not required given the article type (case report).

## Funding

This research did not receive any specific grant from funding agencies in the public, commercial, or not-for-profit sectors.

## CRediT authorship contribution statement

Farzad Omidi-Kashani: Writing - original draft, Conceptualization, Methodology, Formal analysis, Visualization.

Ali Parsa: Resources, Data curation, writing review and editing.

Daniel Madarshahian: Study conception writing - review & editing, data collection, Visualization.

## Guarantor

Farzad Omidi-Kashani.

## Registration of research studies

Not applicable.

## Consent

Written informed consent was obtained from the patient for publication of this case report and accompanying images. A copy of the written consent is available for review by the Editor-in-Chief of this journal on request.

## Provenance and peer review

Not commissioned, externally peer-reviewed.

## Availability of data and materials

The data used to support the findings of this study are available from the corresponding author upon request.

## Declaration of competing interest

The author declares no conflict of interest.
